# Supplementary material for: Effects of PrObiotics on the Symptoms and Surgical ouTComes after Anterior REsection of Colon Cancer (POSTCARE): A Randomized, Double-Blind, Placebo-Controlled Trial
Source: J Clin Med. 2020 Jul 10;9(7):2181. doi: 10.3390/jcm9072181 (PMC7408996; doi:10.3390/jcm9072181)
Supplement: Supplementary file 1 [file jcm-09-02181-s001.pdf]

Supplementary Materials

Table S1. Changes in defecatory function based on responses to the anterior resection syndrome questionnaire.

			**Placebo**	**Probiotics**	***P*-value**		**Placebo**	**Probiotics**	***P*-value**
Flatus	Baseline	No	26 (83.9%)	18 (62.1%)	0.0655				
		Less than 1 per week	3 (9.7%)	3 (10.3%)					
		At least 1 per week	2 (6.5%)	8 (27.6%)					
	Visit 6	No	21 (67.7%)	21 (72.4%)	0.7156	**Worse** than baseline	9 (29.0%)	4 (13.8%)	0.2913
		Less than 1 per week	7 (22.6%)	4 (13.8%)		**Same** as baseline	19 (61.3%)	19 (65.5%)	
		At least 1 per week	3 (9.7%)	4 (13.8%)		**Better** than baseline	3 (9.7%)	6 (20.7%)	
	Visit 7	No	17 (56.7%)	20 (69.0%)	0.6995	**Worse** than baseline	12 (40.0%)	4 (13.8%)	0.0331
		Less than 1 per week	8 (26.7%)	5 (17.2%)		**Same** as baseline	15 (50.0%)	16 (55.2%)	
		At least 1 per week	5 (16.7%)	4 (13.8%)		**Better** than baseline	3 (10.0%)	9 (31.0%)	
Accidental liquid leakage	Baseline	No	22 (70.9%)	21 (72.4%)	0.4966				
		Less than 1 per week	7 (22.6%)	4 (13.8%)					
		At least 1 per week	2 (6.5%)	4 (13.8%)					
	Visit 6	No	15 (48.4%)	20 (69.0%)	0.3240	**Worse** than baseline	10 (32.3%)	6 (20.7%)	0.5943
		Less than 1 per week	12 (38.7%)	7 (24.1%)		**Same** as baseline	16 (51.6%)	18 (62.1%)	
		At least 1 per week	4 (12.9%)	2 (6.9%)		**Better** than baseline	5 (16.1%)	5 (17.2%)	
	Visit 7	No	22 (73.3%)	22 (75.9%)	0.3678	**Worse** than baseline	5 (16.7%)	5 (17.2%)	0.9704
		Less than 1 per week	8 (26.7%)	5 (17.2%)		**Same** as baseline	18 (60.0%)	18 (62.1%)	
		At least 1 per week	0 (0.0%)	2 (6.9%)		**Better** than baseline	7 (23.3%)	6 (20.7%)	
Frequency of defecation	Baseline	More than 7 times per day	1 (3.2%)	1 (3.5%)	0.7538				
		4–7 times per day	1 (3.2%)	3 (10.3%)					
		1–3 times per day	25 (80.7%)	20 (69.0%)					
		Less than 1 per day	4 (12.9%)	5 (17.2%)					
	Visit 6	More than 7 times per day	2 (6.5%)	4 (13.8%)	0.3268				
		4–7 times per day	8 (25.8%)	11 (37.9%)		**Worse** than baseline	10 (32.3%)	14 (48.3%)	0.3787

		1–3 times per day	19 (61.3%)	11 (37.9%)		**Same** as baseline	20 (64.5%)	13 (44.8%)	
		Less than 1 per day	2 (6.5%)	3 (10.3%)		**Better** than baseline	1 (3.2%)	2 (6.90%)	
	Visit 7	More than 7 times per day	2 (6.7%)	3 (10.3%)	0.8846				
		4–7 times per day	9 (30.0%)	7 (24.1%)		**Worse** than baseline	13 (43.3%)	14 (48.3%)	0.7632
		1–3 times per day	17 (56.7%)	18 (62.1%)		**Same** as baseline	16 (53.3%)	13 (44.8%)	
		Less than 1 per day	2 (6.7%)	1 (3.5%)		**Better** than baseline	1 (3.3%)	2 (6.9%)	
Incomplete defecation sensation	Baseline	No	26 (83.9%)	16 (55.2%)	**0.0540**				
		Less than 1 per week	4 (12.9%)	8 (27.6%)					
		At least 1 per week	1 (3.2%)	5 (17.2%)					
	Visit 6	No	11 (35.5%)	13 (44.8%)	0.5708	**Worse** than baseline	16 (51.6%)	9 (30.0%)	0.2078
		Less than 1 per week	9 (29.0%)	5 (17.2%)		**Same** as baseline	14 (45.2%)	17 (58.6%)	
		At least 1 per week	11 (35.5%)	11 (37.9%)		**Better** than baseline	1 (3.2%)	3 (10.3%)	
	Visit 7	No	12 (40.0%)	11 (37.9%)	1.0000	**Worse** than baseline	15 (50.0%)	12 (41.4%)	0.5115
		Less than 1 per week	9 (30.0%)	8 (27.6%)		**Same** as baseline	13 (43.3%)	12 (41.4%)	
		At least 1 per week	9 (30.0%)	10 (34.5%)		**Better** than baseline	2 (6.7%)	5 (17.2%)	
Incomplete defecation sensation	Baseline	No	25 (80.7%)	21 (72.4%)	0.6794				
		Less than 1 per week	4 (12.9%)	4 (13.8%)					
		At least 1 per week	2 (6.5%)	4 (13.8%)					
	Visit 6	No	16 (51.6%)	17 (58.6%)	0.6135	**Worse** than baseline	12 (38.7%)	7 (24.1%)	0.3211
		Less than 1 per week	9 (29.0%)	5 (17.2%)		**Same** as baseline	14 (45.2%)	19 (65.5%)	
		At least 1 per week	6 (19.4%)	7 (24.1%)		**Better** than baseline	5 (16.1%)	3 (10.3%)	
	Visit 7	No	21 (70.0%)	13 (44.8%)	0.1711	**Worse** than baseline	9 (30.0%)	13 (44.8%)	0.4858
		Less than 1 per week	5 (16.7%)	10 (34.5%)		**Same** as baseline	15 (50.0%)	12 (41.4%)	
		At least 1 per week	4 (13.3%)	6 (20.75)		**Better** than baseline	6 (20.0%)	4 (13.8%)	

P-value by Fisher's exact test

Table S2. Comparison of inflammatory markers between the placebo and probiotic groups.

		Placebo (n=31)		Probiotics (n=29)		*P*-value*	*P*-value**	*P*-value***
		n	Mean (±SD)	n	Mean (±SD)			
GPS	Baseline (Visit 2)	31	0.23 (±0.56)	27	0.04 (±0.19)	0.1213		
	Visit 4	31	1.61 (±0.56)	29	1.48 (±0.63)	0.4293	0.9516	0.7417
	Visit 5	30	1.77 (±0.50)	28	1.50 (±0.51)	0.0258	0.4820	0.6187
	Visit 6	31	0.10 (±0.30)	29	0.03 (±0.19)	0.3465	0.3342	0.2733
WBC	Baseline (Visit 2)	31	6.97 (±2.17)	29	6.14 (±1.40)	0.0613		
	Visit 4	31	9.33 (±3.06)	29	9.29 (±2.35)	0.8591	0.2640	0.2621
	Visit 5	31	7.17 (±2.57)	29	6.73 (±1.76)	0.6625	0.4159	0.5358
	Visit 6	31	6.82 (±2.30)	29	5.76 (±1.39)	0.0255	0.5740	0.6031
Neutrophils	Baseline (Visit 2)	31	58.31 (±11.74)	29	58.30 (±8.16)	0.8359		
	Visit 4	30	73.35 (±7.69)	28	75.18 (±6.92)	0.3751	0.4984	0.5168
	Visit 5	31	64.41 (±9.06)	29	65.79 (±9.94)	0.6412	0.5056	0.6412
	Visit 6	31	57.50 (±7.96)	29	52.76 (±9.72)	0.1134	0.1117	0.0601
Lymphocytes	Baseline (Visit 2)	31	31.69 (±9.73)	29	32.31 (±7.18)	0.6896		
	Visit 4	30	17.84 (±6.54)	28	16.40 (±6.03)	0.4692	0.3386	0.3946
	Visit 5	31	23.76 (±7.92)	29	22.91 (±8.68)	0.6359	0.4201	0.5650
	Visit 6	31	31.78 (±7.05)	29	34.91 (±7.88)	0.1430	0.4330	0.2798
NLR	Baseline (Visit 2)	31	2.13 (±1.06)	29	1.95 (±0.73)	0.7843		
	Visit 4	30	4.88 (±2.46)	28	5.45 (±2.61)	0.5034	0.2692	0.2398
	Visit 5	31	3.31 (±2.09)	29	3.75 (±2.94)	0.5892	0.4033	0.3339
	Visit 6	31	2.00 (±1.02)	29	1.64 (±0.65)	0.0771	0.5296	0.3868
Monocytes	Baseline (Visit 2)	31	7.24 (±2.72)	29	6.84 (±1.76)	0.8014		
	Visit 6	31	6.74 (±2.18)	29	6.78 (±1.61)	1.0000	0.8359	0.4863
Plt count	Baseline (Visit 2)	31	259.61 (±84.35)	29	259.76 (±58.96)	0.7561		
	Visit 6	31	324.52 (±96.96)	29	319.97 (±94.36)	0.9646	0.8824	0.8273
Zonulin	Baseline (Visit 2)	31	3.32 (±4.22)	29	4.73 (±4.64)	0.0265		
	Visit 6	31	2.94 (±3.24)	28	2.10 (±1.73)	0.8436	0.0877	0.0353
IFN-γ	Baseline (Visit 2)	31	23.32 (±44.42)	28	11.73 (±7.37)	0.1349		
	Visit 6	31	21.79 (±29.07)	28	13.85 (±7.19)	0.2984	0.5952	0.2202
TNF-α	Baseline (Visit 2)	31	17.15 (±26.72)	28	13.90 (±4.95)	0.3428		

	Visit 6	31	15.10 (±15.07)	28	13.57 (±3.82)	0.2364	0.9939	0.4413
IL-6	Baseline (Visit 2)	31	7.23 (±11.75)	28	4.39 (±2.51)	0.4299		
	Visit 6	31	6.52 (±6.90)	28	4.84 (±2.77)	0.7788	0.7905	0.3752
IL-10	Baseline (Visit 2)	31	38.30 (±48.79)	28	21.57 (±19.09)	0.1429		
	Visit 6	31	38.61 (±31.72)	28	27.72 (±20.89)	0.1290	0.5487	0.1515

*: *P*-value for the comparison of the two groups according to the Wilcoxon rank-sum test.

**: *P*-value for the comparison of the difference in values between the two groups according to the Wilcoxon rank-sum test.

***: *P*-value for group difference according to visit according to generalized estimating equations.

GPS, Glasgow Prognostic Score; WBC, white blood cell; IFN, interferon; TNF, tumor necrosis factor; IL, interleukin; Plt, platelet; NLR, neutrophil-lymphocyte ratio; SD, standard deviation.

Table S3. Operational taxonomic units (OTUs) for analyzed samples in participating patients according to visit.

	**01-Hospital**				**02-Hospital**				**03-Hospital**				**04-Hospital**			
	**Visit 2**	**Visit 3**	**Visit 6**	**Visit 7**	**Visit 2**	**Visit 3**	**Visit 6**	**Visit 7**	**Visit 2**	**Visit 3**	**Visit 6**	**Visit 7**	**Visit 2**	**Visit 3**	**Visit 6**	**Visit 7**
R001	550	1510	367	856	840	1377	601	928	729	1117	786	1295	805	1426	590	1186
R002	686	1159	791	762	632	871	379	-	-	-	-	-	-	-	-	-
R003	596	961	1063	342	612	1022	407	866	646	-	650	1346	941	1260	843	843
R004	419	831	443	743	-	-	-	-	643	1088	624	1167	1332	320	704	1082
R005	1042	1226	560	773	-	-	-	-	726	-	869	474	750	1051	664	813
R006	755	826	940	1432	994	1060	806	924	1120	1151	804	957	783	989	321	658
R007	894	1042	572	710	821	921	1300	1202	504	705	544	421	1035	750	579	1611
R008	662	1222	751	750	1101	1236	966	1083	706	753	866	466	1186	876	735	1134
R009	675	1118	720	546					1030	1158	-	642	-	-	-	-
R010	1082	-	883	845					2283	1523	989	706	1090	1069	1227	1201
R011	690	912	654	616					515	324	530	608	1049	1185	872	1017
R012	768	1152	530	941					1164	1040	1134	1309	592	672	828	855
R013	287	537	488	488					1173	1218	866	726	-	-	-	-
R014	1097	577	1031	811					770	669	190	400	942	735	1041	1185
R015	-	-	-	-					849	-	857	775				
R016	1394	1585	699	690					893	922	728	692				
R017	1086	1152	853	951					459	685	968	899				
R018	-	-	-	-					1240	-	699	1047				
R019	506	716	1167	1131					527	727	592	587				
R020	827	926	760	905					1096	1198	598	364				
R021	1766	1043	645	428					1040	1560	712	561				
R022	1145	1242	553	165					1047	1257	1210	863				
R023									-	1353	1098	1205				

Figure S1. Compositional changes in the phylum level of bacteria in both groups according to visit.

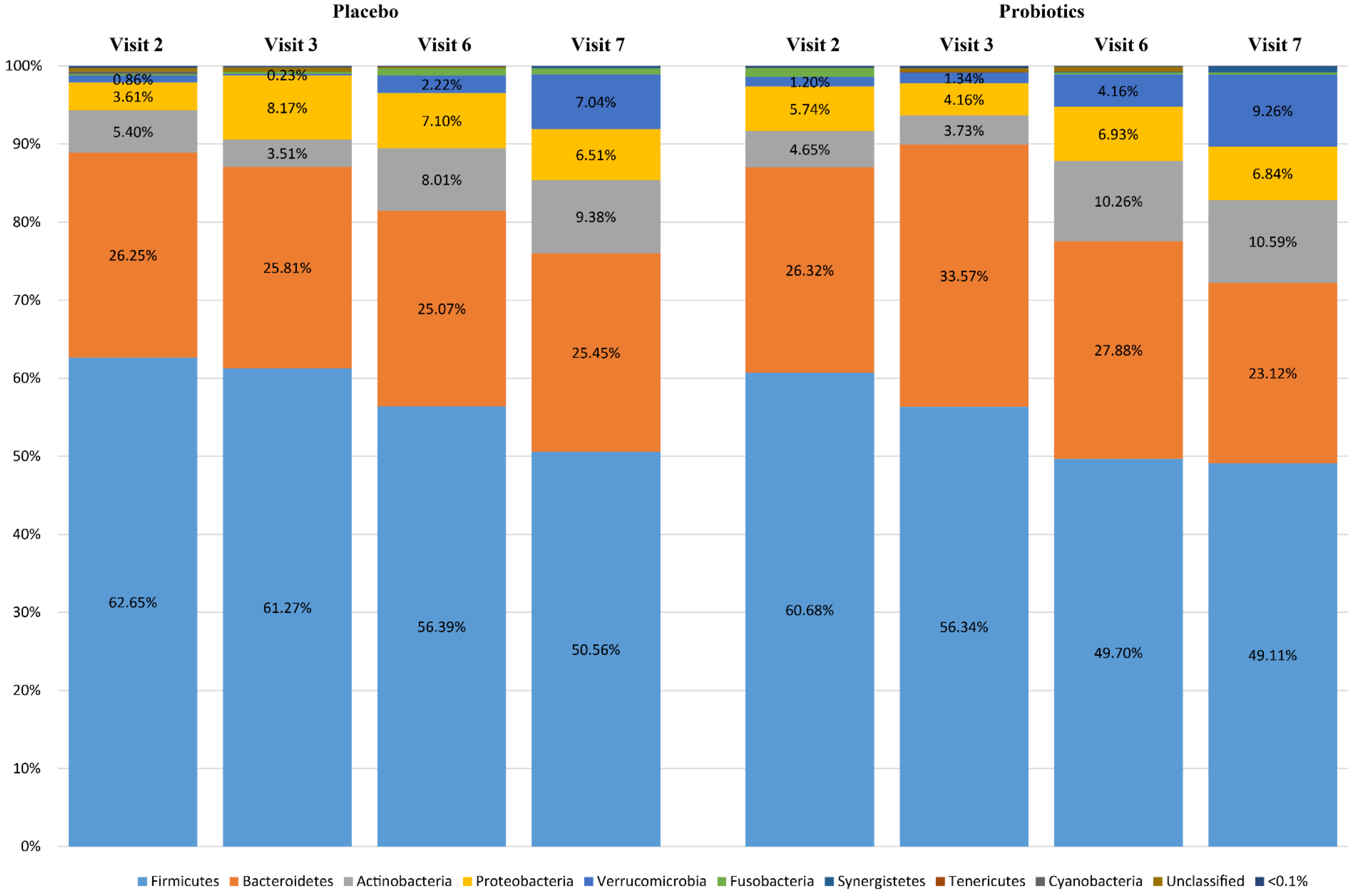
Placebo
Probiotics
Visit 2
Visit 3
Visit 6
Visit 7
Visit 2
Visit 3
Visit 6
Visit 7
100%
90%
80%
70%
60%
50%
40%
30%
20%
10%
0%
0.86%
3.61%
5.40%
26.25%
62.65%
0.23%
8.17%
3.51%
25.81%
61.27%
2.22%
7.10%
8.01%
25.07%
56.39%
7.04%
6.51%
9.38%
25.45%
50.56%
1.20%
5.74%
4.65%
26.32%
60.68%
1.34%
4.16%
3.73%
33.57%
56.34%
4.16%
6.93%
10.26%
27.88%
49.70%
9.26%
6.84%
10.59%
23.12%
49.11%
Firmicutes
Bacteroidetes
Actinobacteria
Proteobacteria
Verrucomicrobia
Fusobacteria
Synergistetes
Tenericutes
Cyanobacteria
Unclassified
<0.1%

Figure S2. Compositional changes in the genus of bacteria in both groups according to visits.

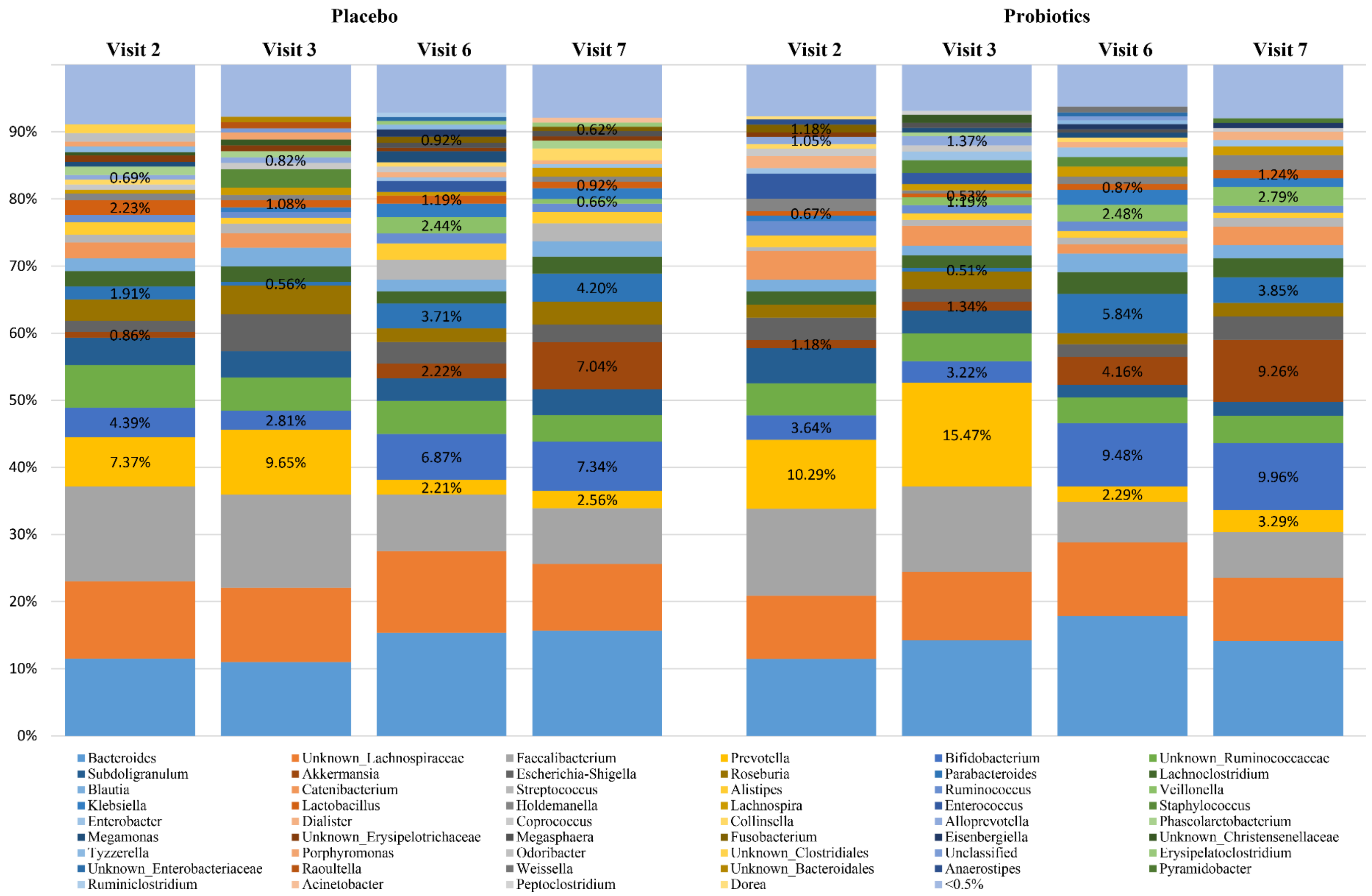

Figure S3. Correlation between metabolites and analyzed bacteria in each group.

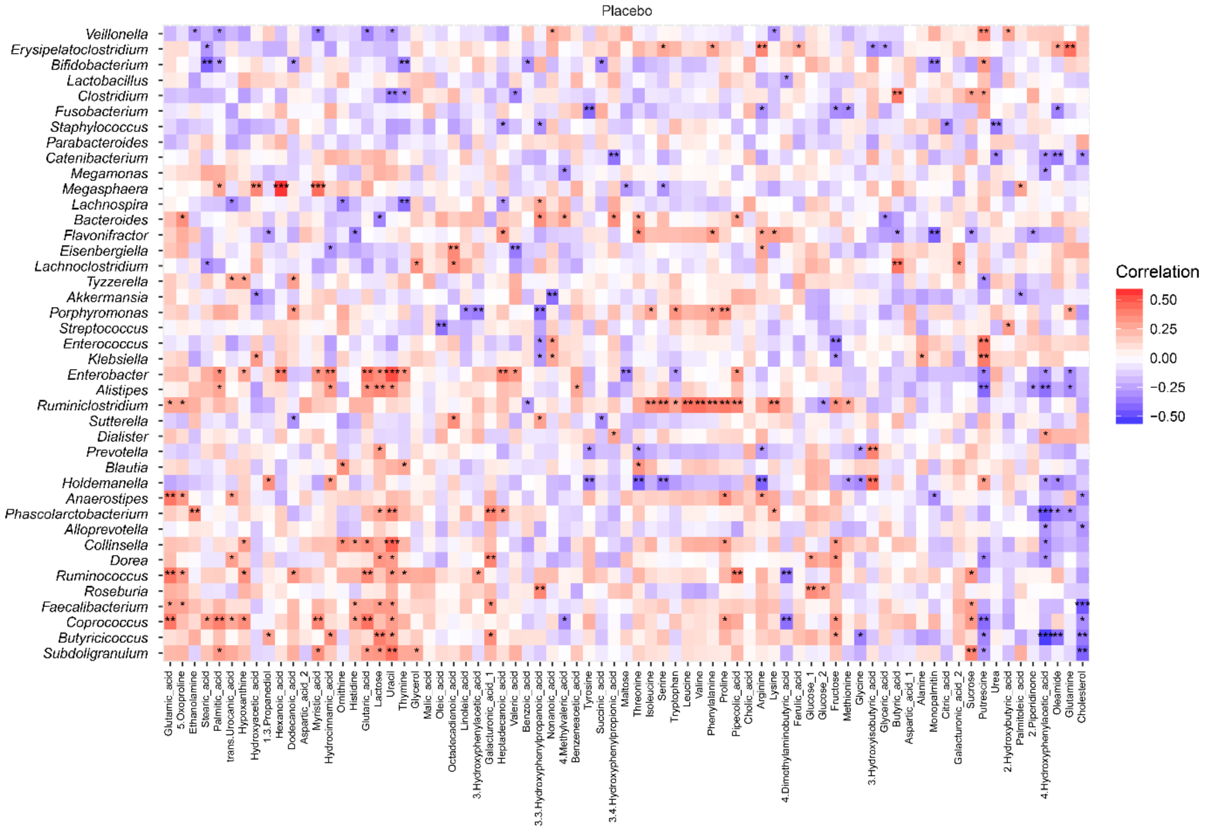

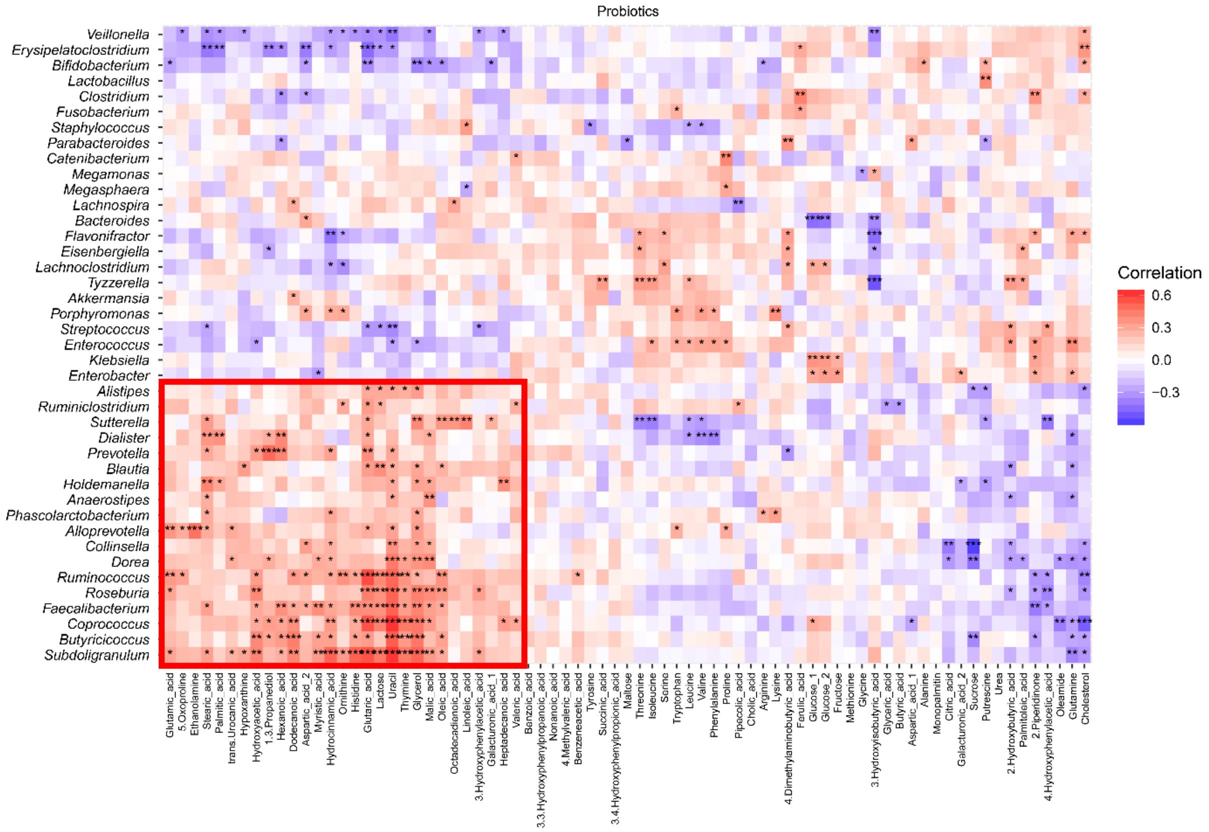
Probiotics
Veillonella
Erysipelatoclostridium
Bifidobacterium
Lactobacillus
Clostridium
Fusobacterium
Staphylococcus
Parabacteroides
Catenibacterium
Megamonas
Megasphaera
Lachnospira
Bacteroides
Flavonifractor
Eisenbergiella
Lachnoclostridium
Tyzzerella
Akkermansia
Porphyromonas
Streptococcus
Enterococcus
Klebsiella
Enterobacter
Alistipes
Ruminiclostridium
Sutterella
Dialister
Prevotella
Blautia
Holdemanella
Anaerostipes
Phascolarctobacterium
Alloprevotella
Collinsella
Dorea
Ruminococcus
Roseburia
Faecalibacterium
Coprococcus
Butyricicoccus
Subdoligranulum
Glutamic_acid
5.Oxoproline
Ethanolamine
Stearic_acid
Palmitic_acid
trans.Urocanic_acid
Hypoxanthine
Hydroxyacetic_acid
1.3.Propanediol
Hexanoic_acid
Dodecanoic_acid
Aspartic_acid_2
Myristic_acid
Hydrocinnamic_acid
Ornithine
Histidine
Glutaric_acid
Lactose
Uracil
Thymine
Glycerol
Malic_acid
Oleic_acid
Octadecadienoic_acid
Linoleic_acid
3.Hydroxyphenylacetic_acid
Galacturonic_acid_1
Heptadecanoic_acid
Valeric_acid
Benzoic_acid
3.3.Hydroxyphenylpropanoic_acid
Nonanoic_acid
4.Methylvaleric_acid
Benzeneacetic_acid
Tyrosine
Succinic_acid
3.4.Hydroxyphenylpropionic_acid
Maltose
Threonine
Isoleucine
Serine
Tryptophan
Leucine
Valine
Phenylalanine
Proline
Pipecolic_acid
Cholic_acid
Arginine
Lysine
4.Dimethylaminobutyric_acid
Ferulic_acid
Glucose_1
Glucose_2
Fructose
Methionine
Glycine
3.Hydroxyisobutyric_acid
Glyceric_acid
Butyric_acid
Aspartic_acid_1
Alanine
Monopalmitin
Citric_acid
Galacturonic_acid_2
Sucrose
Putrescine
Urea
2.Hydroxybutyric_acid
Palmitoleic_acid
2.Piperidinone
4.Hydroxyphenylacetic_acid
Oleamide
Glutamine
Cholesterol
Correlation
0.6
0.3
0.0
−0.3
